# Inositol in Disease and Development: Roles of Catabolism via *myo*-Inositol Oxygenase in *Drosophila melanogaster*

**DOI:** 10.3390/ijms24044185

**Published:** 2023-02-20

**Authors:** Altagracia Contreras, Melissa K. Jones, Elizabeth D. Eldon, Lisa S. Klig

**Affiliations:** 1 Department of Biological Sciences, California State University Long Beach, Long Beach, CA 90840, USA; 2 Department of Biology, Johns Hopkins University, Baltimore, MD 21218, USA; 3 Genentech, South San Francisco, CA 94080, USA

**Keywords:** inositol, metabolism, developmental defect, diabetes, obesity, *Drosophila*, oxygenase, head, proboscis

## Abstract

Inositol depletion has been associated with diabetes and related complications. Increased inositol catabolism, via *myo*-inositol oxygenase (MIOX), has been implicated in decreased renal function. This study demonstrates that the fruit fly *Drosophila melanogaster* catabolizes *myo*-inositol via MIOX. The levels of mRNA encoding MIOX and MIOX specific activity are increased when fruit flies are grown on a diet with inositol as the sole sugar. Inositol as the sole dietary sugar can support *D. melanogaster* survival, indicating that there is sufficient catabolism for basic energy requirements, allowing for adaptation to various environments. The elimination of MIOX activity, via a *piggyBac* WH-element inserted into the *MIOX* gene, results in developmental defects including pupal lethality and pharate flies without proboscises. In contrast, RNAi strains with reduced levels of mRNA encoding MIOX and reduced MIOX specific activity develop to become phenotypically wild-type-appearing adult flies. *myo*-Inositol levels in larval tissues are highest in the strain with this most extreme loss of *myo*-inositol catabolism. Larval tissues from the RNAi strains have inositol levels higher than wild-type larval tissues but lower levels than the *piggyBac* WH-element insertion strain. *myo*-Inositol supplementation of the diet further increases the *myo*-inositol levels in the larval tissues of all the strains, without any noticeable effects on development. Obesity and blood (hemolymph) glucose, two hallmarks of diabetes, were reduced in the RNAi strains and further reduced in the *piggyBac* WH-element insertion strain. Collectively, these data suggest that moderately increased *myo*-inositol levels do not cause developmental defects and directly correspond to reduced larval obesity and blood (hemolymph) glucose.

## 1. Introduction

Alterations in *myo*-inositol metabolism are often associated with human diseases including diabetes, cancer, reproductive defects, and neurological disorders [1,2,3,4,5,6,7,8]. The depletion of *myo*-inositol has been associated with diabetic complications such as nephropathies, cataracts, retinopathies, and neuropathies [9,10,11,12]. Inositol depletion could arise from increased *myo-*inositol catabolism. Rats with upregulated *myo-*inositol catabolism have increased blood glucose and related pathobiological stress [13,14,15]. In the fruit fly, *Drosophila melanogaster,* reduced *myo-*inositol synthesis was shown to cause defective spermatogenesis [16], and increased *myo-*inositol synthesis caused severe developmental defects [17]. This led to the current study investigating the role of *myo-*inositol catabolism in fruit fly development and metabolism. 

Inositol is a six-carbon sugar alcohol found in all eukaryotes and some prokaryotes. It can serve as a precursor of the membrane lipid phosphatidylinositol, act as a second messenger in signal transduction pathways, aid in osmoregulation, mediate endoplasmic reticulum stress (unfolded protein response), affect nucleic acid synthesis, or function as a carbon and/or energy source [18,19,20,21,22,23]. *myo*-Inositol oxygenase (MIOX) catalyzes the first step of *myo-*inositol catabolism and is essential in the regulation of *myo-*inositol levels in vivo [24,25,26,27]. MIOX was first reported in rat kidney extracts, and later in oat seedlings, hog and human kidney, many plants, and yeast [13,25,28,29,30,31,32,33,34,35]. In most organisms, MIOX is an approximately 33 kDa monomeric single-domain protein [36,37]. This highly conserved enzyme catalyzes the ring cleavage of *myo-*inositol with the incorporation of oxygen, converting *myo-*inositol into D-glucuronic acid. D-glucuronic acid can enter in multiple metabolic pathways, including the step-wise conversion into D-xylulose-5-phosphate and then the pentose phosphate cycle, eventually producing nucleic acids and providing energy [25,26,28,29]. The glucuronate–xylulose pathway has been documented as the only *myo*-inositol catabolic pathway in eukaryotes [25].

In recent years, *D. melanogaster* has emerged as an ideal model organism for studying metabolic diseases including diabetes [38,39,40,41,42,43,44,45,46]. It has also been shown, via established assays, to display a wide array of diabetic-like traits similar to humans such as increased circulating glucose, insulin resistance, excess lipid storage, and decreased longevity [38,39,42,45,47,48,49,50,51,52,53,54,55,56]. The development of the fruit fly consists of gametic, embryonic, larval, pupal, and adult stages. During embryogenesis, the imaginal disc primordia are established, and head involution occurs. Head involution includes the rearrangement of lobes that form larval head structures, concurrent with the relocation of the imaginal disc primordia that later contribute to adult head structures, including the proboscis [57,58]. Rivera et al. [17] demonstrated that dietary *myo-*inositol and/or increasing *myo-*inositol synthesis via genetic manipulation alleviated obesity and high-hemolymph glucose; however, extremely high levels of constitutive *myo-*inositol synthesis resulted in pupal lethality and developmental defects (lacking proboscises and with structural alterations of the legs and wings).

In the current study, *myo-*inositol catabolism and its role in growth, development, and adaptation to varied environments were explored in the model organism *D. melanogaster*. *D. melanogaster* were shown to survive with *myo-*inositol as the sole dietary sugar. Moreover, this study seems to be the first demonstration in animals that *MIOX* mRNA levels and MIOX specific activity levels are regulated in response to dietary *myo-*inositol. A *piggyBac* WH-element insertion strain, with MIOX specific activity eliminated, displayed high levels of pupal lethality and pharate adult developmental defects (no proboscises). Third-instar larvae of three independent *D. melanogaster* strains with reduced *MIOX* mRNA levels and reduced MIOX specific activity levels were shown to have a dramatic reduction in obesity and high-hemolymph glucose. Lastly, genetic modifications cause high levels of *myo-*inositol mitigate diabetic traits but display developmental defects, while dietary *myo-*inositol supplementation mitigates diabetic traits without inducing developmental defects. These studies contribute to the understanding of the role of *myo-*inositol in metabolism and development.

## 2. Results

### 2.1. MIOX Homolog Identified in D. melanogaster (CG6910) Is Regulated in Response to Dietary myo-Inositol 

**Figure 1:**
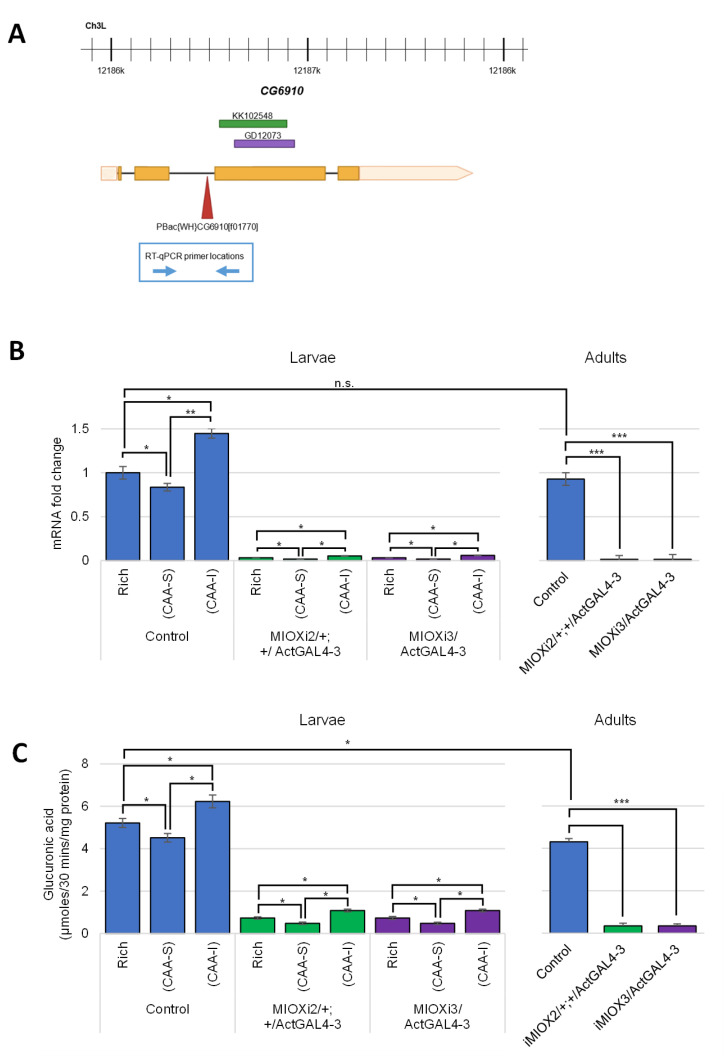
***D.****melanogaster MIOX (CG6910*) is regulated in response to dietary *myo-*inositol. (**A**) The *D. melanogaster myo-*inositol catabolic gene (*myo-*inositol oxygenase) *CG6910*, the surrounding genomic region of chromosome 3, and the transcript (isoform B) is displayed. The 5′ and 3′ UTRs are peach, and the exons are orange. The locations of the *MIOXi2* (KK102548, green) and *MIOXi3* (GD12073, purple) sequences and the ~7.2 kb *piggyBac* WH-element insertion (red) are also displayed. The primer locations for the qRT-PCR experiments are blue arrows. (**B**) qRT-PCR experiments examining *MIOX* mRNA levels of larvae (left) and adults (right) grown on rich or semi-defined sucrose (CAA-S) or semi-defined *myo-*inositol (CAA-I) food. Normalized to *RpL32*, mean ± SE of three independent trials are represented. Control strains *ActGal4-3*/*TbGFP* and *CyOGFP*/*+*; *ActGal4-3*/*+* were indistinguishable from the wild-type control Canton-S results. n.s. = not significant, * *p* < 0.05, ** *p*< 0.005, *** *p* < 0.0001 as indicated, determined by two-tailed *t*-test. (**C**) MIOX enzyme assays to determine *myo-*inositol oxygenase specific activity in crude lysates of larvae and adults (as indicated) grown on rich, CAA-S, or CAA-I food. Mean ± SE of three independent trials are represented. There was no detectable MIOX activity in crude lysates of the homozygous *piggyBac* WH-element insertion strain (P-*miox*^f01770^/P-*miox*^f01770^). Control strains *ActGal4-3*/*TbGFP* and *CyOGFP*/*+*: *ActGal4-3*/*+* were indistinguishable from the wild-type control Canton-S results shown. * *p* < 0.05, *** *p* < 0.0001 as indicated, determined by two-tailed *t*-test.

To identify *myo-*inositol oxygenase (MIOX) in *Drosophila melanogaster*, BLASTP was performed using the 285 amino acid human MIOX protein sequence as a query. This revealed one candidate (*CG6910*) with ~55% identity spanning the entire protein. Although there are two splice variants of *CG6910,* B and C, listed in Flybase [59], 5′RACE experiments previously performed in this lab did not detect the C variant in larvae or adults. Moreover, high-throughput expression data (RNA -Seq Signal by Region) and the G-browse visual display reveal that RNA transcripts unique to the C variant region are rare or undetectable in all stages of development and in all tissues examined [59]. To experimentally verify that *CG6910* encoded the MIOX protein, three strains were obtained; two RNAi strains to reduce *CG6910* transcript levels and one strain with a *piggyBac* WH-element inserted into *CG6910* (Figure 1A). 

To determine if the levels of *CG6910* (*MIOX*) mRNA are regulated in response to *myo-*inositol, qRT-PCR experiments were performed. RNA was extracted from third-instar larvae and adults of five *D. melanogaster* strains. Two of the strains have different *MIOX* (*CG6910*) RNAi constructs with UAS_GAL4_ sequences. Both are controlled by GAL4 driven by the Actin 5C (Act5C) promoter (*MIOXi2*/*+*; *+*/*Act5CGal4-3* and *MIOXi3*/*Act5CGal4-3).* Three control strains were also used (CS, *ActGal4-3*/*Tb*, and *CyOGFP*/*+*; *ActGal4-3*/*Tb*). The adults had slightly lower *MIOX* mRNA levels than the larvae. *MIOX* (*CG6910)* mRNA levels in *MIOXi2*/*+*; *+*/*Act5Cgal4-3* and *MIOXi3*/*Act5CGal4-3* were significantly lower than in the control strains. When larvae were grown on semi-defined food with *myo-*inositol as the sole sugar (CAA-I), the level of *MIOX* mRNA was significantly higher than when grown on semi-defined food with sucrose as the sole sugar (CAA-S) (Figure 1B).

To examine if dietary *myo-*inositol affected MIOX specific activity, the conversion of *myo-*inositol to glucuronic acid by crude lysates of third-instar larvae and adults was monitored in the strains described above. There was slightly less activity detectable in the adults than the larvae. MIOX specific activity in larvae was significantly higher for all the strains when grown on CAA-I. Moreover, the specific activity of MIOX in crude lysates of *MIOXi2*/*+, +*/*Act5CGal4-3,* and *MIOXi3*/*Act5CGal4-3* larvae was significantly lower than that of the control larvae (Figure 1C). Even more striking is that there was no detectable MIOX activity in crude lysates of the homozygous *piggyBac* WH-element insertion strain (P-*miox*^f01770^/P-*miox*^f01770^). 

### 2.2. Dietary myo-Inositol Supports Survival of Wild-Type (CS) D. melanogaster Adults but Not of MIOX Knockdown Strains

**Figure 2:**
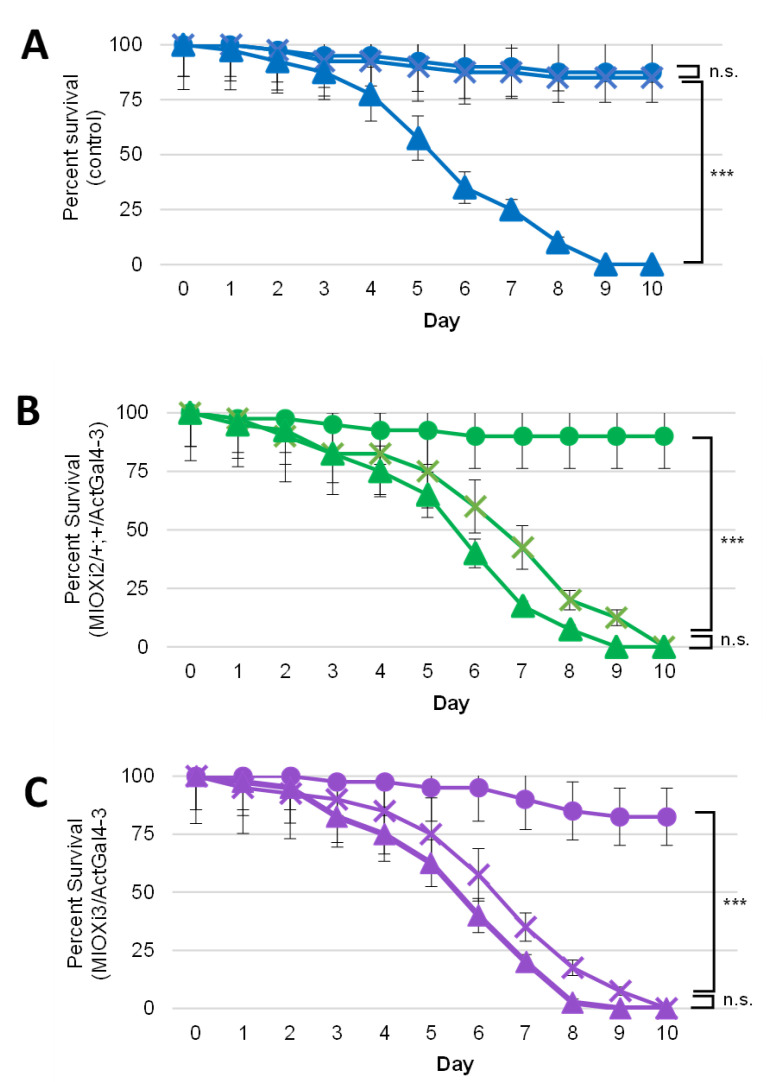
Wild-type (CS) *D. melanogaster* adults, but not *MIOX*i*2*/*+*; *+*/*Act5CGal4-3 and MIOXi3*/*Act5CGal4-3* adults, are viable on food with *myo-*inositol as the sole sugar (CAA-I). On CAA-S, all the strains survive, and without sugar (CAA-0), all the strains die. Survivals on three foods are displayed: CAA-S (●), CAA-I (x), and CAA-0 (▲). (**A**) Wild-type control strain Canton S. (**B**) *MIOX*i2/+; +/Act5CGal4-3. (**C**) *MIOX*i3/Act5CGal4-3. Mean ± SE of six independent trials with twenty flies (half male/half female) per trial. n.s. = not significant, *** *p* < 10^−6^ as indicated determined by Mantel–Cox (log rank) test.

To determine if MIOX plays a role in fruit fly survival, pupae of *MIOXi2*/*+*; *+*/*Act5CGal4-3, MIOXi3*/*Act5CGal4-3,* and the wild-type control strain (CS) were transferred to tubes with semi-defined food containing sucrose (CAA-S) or *myo-*inositol as the sole sugar (CAA-I), or no sugar (CAA-0). It was exciting to note that wild-type (CS) adult flies survived equally well on semi-defined food with either *myo-*inositol or sucrose as the sole sugar, demonstrating that there is sufficient *myo-*inositol catabolism to support survival of *D. melanogaster* (Figure 2A).

Moreover, control, *MIOXi2*/*+*; *+*/*Act5CGal4-3,* and *MIOXi3*/*Act5CGal4-3* adults survived comparably well on semi-defined food with sucrose (CAA-S). On semi-defined no-sugar food (CAA-0), all three strains died within ten days (Figure 2A–C). In contrast to wild-type flies, *MIOXi2*/*+*; *+*/*Act5CGal4-3* and *MIOXi3*/*Act5CGal4-3* adults died by day 10 on semi-defined food with *myo-*inositol as the sole sugar (CAA-I). The survival of these two strains on CAA-I was similar to their survival on food with no-sugar (CAA-0) (Figure 2B,C). 

### 2.3. Disruption of myo-Inositol Catabolism via Piggybac WH-Element Insertion in MIOX Results in Developmental Defects

**Figure 3:**
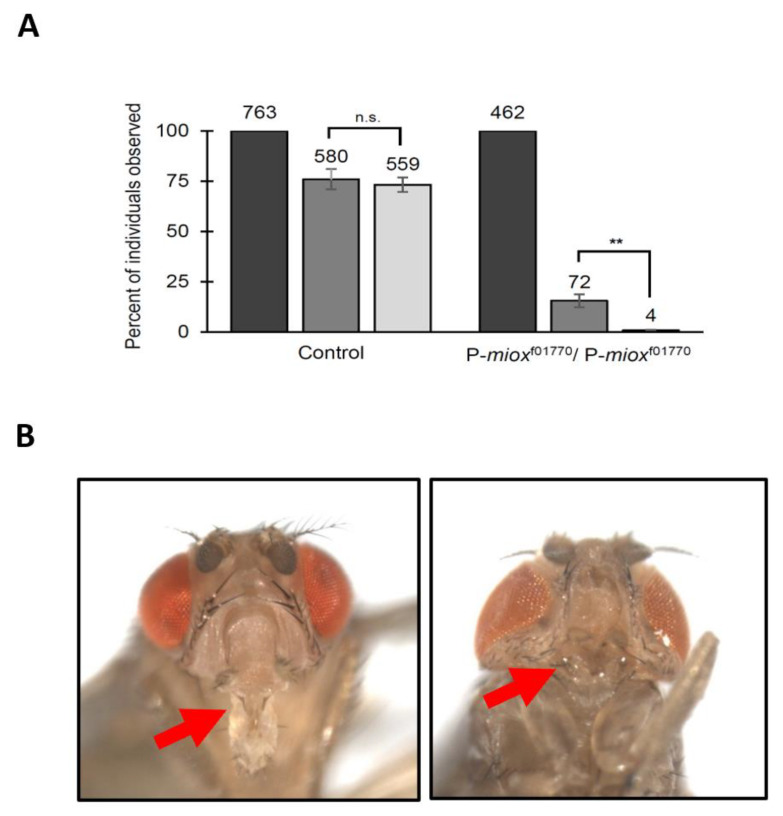
Disruption of *myo-*inositol catabolism via *piggyBac* WH-element insertion in *MIOX* results in developmental defects. (**A**) The percent of adults (light grey bars) eclosing from pupae (medium grey bars), normalized to the number of embryos (dark grey bars) on standard rich food. Strains as indicated. Wild-type control (CS) results shown. N = total number of individuals examined. Mean ± SE of three independent trials are represented. n.s. = not significant, ** *p* < 0.005, as determined by two-tailed *t*-test. (**B**) Brightfield microscope images of adult flies after eclosion. On the left is the control heterozygous P-*miox*^f01770^/TbGFP (N = 15) (indistinguishable from the wild-type control Canton-S), and on the right is P-*miox*^f01770^/P-*miox*^f01770^ (N = 16). The arrows indicate the proboscis in the wild-type or the region lacking the proboscis in the *piggyBac* WH-element insertion strain.

The survival experiments described above did not include adult P-*miox*^f01770^/P-*miox*^f01770^ flies, because these homozygotes are not viable as adults. As displayed in Figure 3A, ~16% of the homozygous P-*miox*^f01770^/P-*miox*^f01770^ and ~76% of the control embryos developed to the pupal stage. Even more striking is that only ~6% of the homozygous P-*miox*^f01770^/P-*miox*^f01770^ pupae eclosed as adults, with most dying as pharate adults (not eclosing from the pupal case), in contrast to ~96% of the control strain eclosing as adults. The few P-*miox*^f01770^/P-*miox*^f01770^ adults that eclosed from the pupal case died within two days and exhibited severe head morphological defects, most noticeably the lack of a proboscis (Figure 3B). To confirm that the *piggyBac* WH-element in P-*miox*^f01770^ caused the pupal lethality and morphological defect, heterozygous strains with the element excised were generated. All three independently obtained excision lines (eleven stocks), with TM6, Tb balancer chromosomes, produced viable homozygous progeny in the expected ratio. Of the 998 pupae examined (350 homozygotes; 648 heterozygotes (Tb)), the same percentage of homozygotes and heterozygotes eclosed. Neither pupal lethality nor morphological defects were observed in the homozygous excision progeny. The excision of the element reverted the phenotype.

### 2.4. Reduced myo-Inositol Catabolism Increases myo-Inositol Levels in Larvae

**Figure 4:**
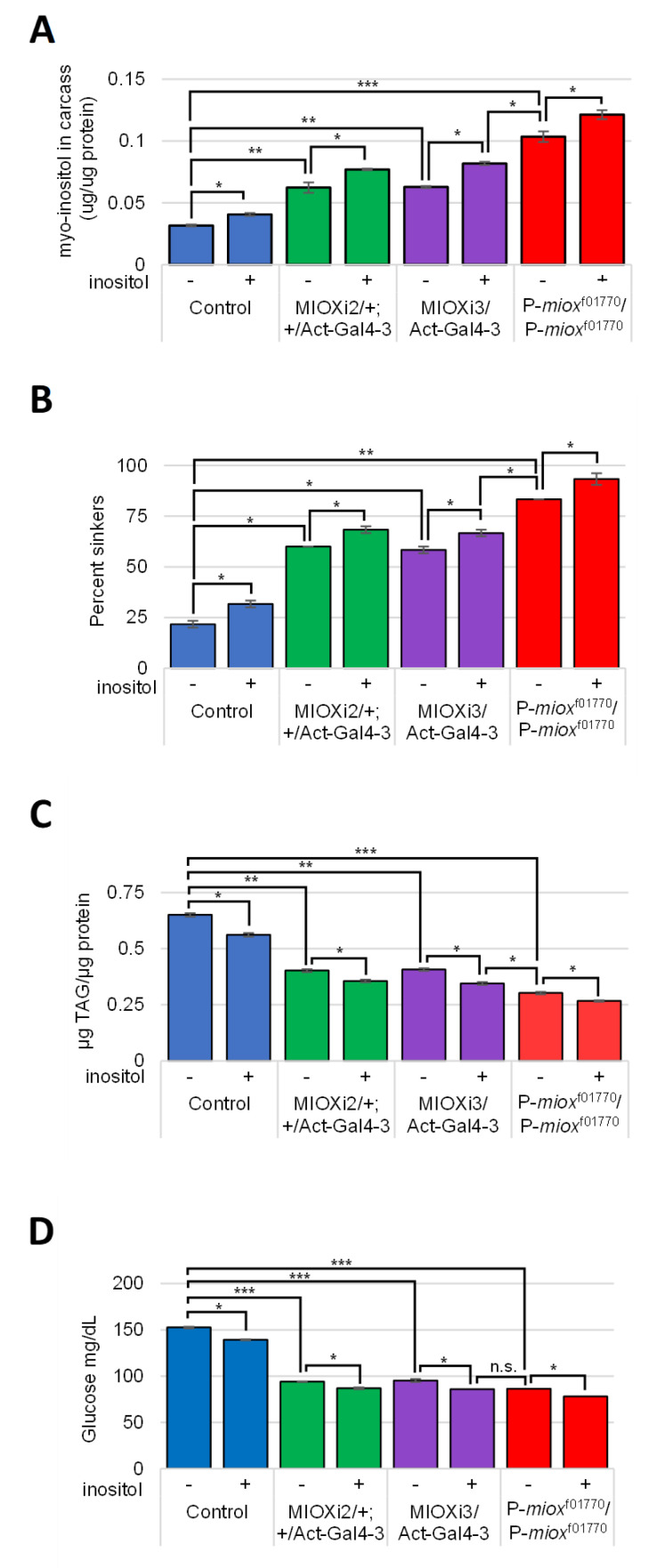
Reduced *myo-*inositol catabolism increases *myo-*inositol levels but decreases larval obesity and hemolymph glucose. Larvae grown on standard rich food with *myo-*inositol supplementation as indicated. (**A**) Larval carcasses assayed for *myo-*inositol; values indicated are normalized to total protein. N = 5 per condition per trial. (**B**) Buoyancy assay; the percentage of larvae that sink are displayed. N = 20 per condition per trial. (**C**) TAG assay; values indicated are normalized to total protein. N = 6 per condition per trial. (**D**) Glucose (mg/dL) assay of hemolymph. N = 5 per condition per trial. Mean ± SE of three independent trials of each experiment are represented. * *p* < 0.05; ** *p* < 0.005; *** *p* < 0.0001 as indicated, determined by two-tailed *t*-test. Control strains ActGal4-3/TbGFP and CyOGFP/+: ActGal4-3/+ were indistinguishable from the wild-type control Canton-S results shown for all four experiments.

To assess if the developmental defect observed in the P-*miox*^f01770^/P-*miox*^f01770^ strain was due to reduced catabolism yielding increased *myo-*inositol levels, assays were performed using carcasses of third-instar larvae grown on rich food with 0 or 50 µM *myo-*inositol supplementation. Higher *myo-*inositol levels were observed in the tissues of larvae with decreased *myo-*inositol catabolism (Figure 4A). The highest level of *myo-*inositol is apparent in the P-*miox*^f01770^/P-*miox*^f01770^ larval tissues which had no detectable *myo-*inositol catabolic activity via MIOX. In all the strains, *myo*-inositol levels increased when the standard rich food was supplemented with 50 µM of *myo-*inositol (Figure 4A).

### 2.5. Increased myo-Inositol Decreases Larval Obesity and Hemolymph Glucose

To examine the relationship between MIOX and the diabetic hallmarks, obesity and high hemolymph glucose, third-instar larvae grown on standard rich food with 0 or 50 µM *myo-*inositol supplementation were assayed. Buoyancy, TAG, and glucose assays revealed that *P*-*miox*^f01770^/P-*miox*^f01770^ larvae were the least obese with the lowest levels of TAG and hemolymph glucose. In these assays, the two RNAi knockdown strains, *MIOXi2*/*+*; *+*/*Act5CGal4-3* and *MIOXi3*/*Act5CGal4-3,* had intermediate levels relative to the control and the P-*miox*^f01770^/P-*miox*^f01770^ strains (Figure 4B–D). Dietary *myo-*inositol supplementation (50 µM) further reduced the proportion of obese larvae, TAG, and hemolymph glucose in all strains (Figure 4B–D). 

## 3. Discussion

This study examines the roles of *myo-*inositol catabolism using the model organism *D. melanogaster*. *CG6910* was identified as the *myo-*inositol catabolic gene encoding *myo-*inositol oxygenase (MIOX), which is more than 55% identical (>70% similar) to human MIOX. The high level of identity among these two evolutionarily distant organisms demonstrates the conservation of MIOX structures in eukaryotes. There are two splice variants, B and C, listed in Flybase [59]; however, multiple experiments suggest that RNA transcripts unique to the C variant region are rare or undetectable in all stages of development and in all tissues examined [59]. If the C variant exists in larvae and adults, it comprises a small proportion of the MIOX transcripts and protein. Developmental proteome experiments reveal high levels of MIOX expression in late third-instar larvae (wandering and prepupae) and adults [60]. Temporal microarray and RNAseq data have shown the peak expression of *MIOX* (*CG6910*) mRNA in late third-instar larvae and adults [61]; therefore, an emphasis has been placed on examining third-instar larvae and adults.

This appears to be the first report to examine *MIOX* (*CG6910*) mRNA levels in animals, *D. melanogaster* larvae and adults, in response to dietary *myo-*inositol (Figure 1B). A significantly higher level of *MIOX* mRNA is apparent in qRT-PCR experiments examining larvae fed *myo-*inositol as the sole sugar (CAA-I) compared to larvae fed sucrose as the sole sugar (CAA-S). *MIOX* mRNA levels were reduced in larvae and adults via two different RNAi constructs (*MIOXi2*/*+*; *+*/*Act5CGal4-3* and *MIOXi3*/*Act5CGal4-3*) (Figure 1B). Similar to the wild-type control, these strains have higher levels of MIOX mRNA when grown on CAA-I than when grown on CAA-S (Figure 1B). Increased *myo-*inositol catabolism, via MIOX, has been implicated in decreased renal function [62]. Decreased *MIOX* mRNA levels via siRNA in transgenic mice expressing high levels of *MIOX* mRNA has been shown to reduce renal damage and associated endoplasmic reticulum stressors [63]. Yet, in this study using *D. melanogaster*, neither the RNAi strain nor the wild-type controls exhibited any gross morphological abnormalities, even with increased levels of *MIOX* mRNA when fed CAA-I. 

An assay to measure MIOX-specific activity in *D. melanogaster* was established based on previously existing protocols for rat kidneys, hog kidneys, and fungi [28,31,32] (Figure 1C). Similar to the mRNA levels, MIOX-specific activity levels in *MIOXi2*/*+*; *+*/*Act5CGal4-3* and *MIOXi3*/*Act5CGal4-3* larvae and adults were lower than the control (CS) strain. Moreover, all the strains had increased levels of MIOX activity when fed CAA-I. Homozygous *CG6910*-*MIOX* (P-*miox*^f01770^/P-*miox*^f01770^) larvae with a 7.2 kb *piggyBac* WH-element insertion disrupting the *MIOX* gene had no detectable MIOX activity. The MIOX-specific activity in crude lysates of control *D. melanogaster* larvae is 5.2 µmol/30 mins/mg, slightly more than that observed in adults, much more than that in rat kidney [29], and similar to that in the fungus *Cryptococcus neoformans* [32].

Survival experiments revealed that adult *D. melanogaster* have sufficient *myo-*inositol catabolism and transport to remain viable on semi-defined food with *myo-*inositol as the sole sugar/energy source (CAA-I) (Figure 2). The statistically significant results of six independent trials also showed that a reduction in *MIOX* expression via RNAi diminishes viability on CAA-I, mimicking survival on food with no sugar. The identical loss of the viability of both fly strains, *MIOXi2*/*+*; *+*/*Act5CGal4-3* and *MIOXi3*/*Act5CGal4-3,* is particularly compelling, because the RNAi constructs are located on separate chromosomes and the strains were generated from two separate Vienna Drosophila Resource Center (VDRC) libraries (KK (phiC31) and GD (P-element), respectively). These libraries used different vectors and cloning methods and have been shown to have different off-target effects [64,65,66,67]. Since the two strains appeared phenotypically identical, the observed phenotypes in this study should be due to the decreased *MIOX* mRNA levels. Moreover, these results indicate that MIOX is a component of the primary *myo-*inositol catabolic pathway in *D. melanogaster.*

Adult P-*miox*^f01770^/P-*miox*^f01770^ were not included in experiments because they are rarely viable, with only ~6% of the pupae eclosing and the flies that eclose dying within two days (Figure 3A). These results are similar to previously published findings that highly upregulated *myo*-inositol synthesis reduces eclosion to ~9% [17]. As *myo-*inositol is a precursor of the phosphatidylinositol phosphates (PIPs), it is interesting to note that inositol phosphate kinase 2 (*Ipk2*) deletions and dysregulation of the expression of the phosphatidylinositol synthase gene (*Pis*) also cause pupal lethality [68,69]. Among the few P-*miox*^f01770^/P-*miox*^f01770^ adults, the most jarring morphological defect is the lack of proboscises (Figure 3B). This phenotype has been previously described when *myo-*inositol synthesis was highly upregulated [17]. The *MIOX* RNAi knockdown strains with intermediate levels of of *myo*-inositol in larval tissues did not display morphological abnormalities, paralleling previously published findings that lower but still elevated levels of *myo*-inositol synthesis did not produce the developmental defect [17]. Together, these data suggest that increased *myo-*inositol, not the process of synthesis or catabolism, contributes to or causes developmental defects. Deformities of fruit fly head structures have been observed with mutations disrupting the *head involution defective* (*hid*) [70,71] or the *decapentaplegic* (*dpp*) genes [72,73,74]. The morphological abnormalities observed in this study, however, seem to be unique to flies with elevated *myo-*inositol levels. 

As could be predicted, less *myo-*inositol catabolism results in more *myo-*inositol in larval tissues, with the highest *myo-*inositol level observed in the strain with the lowest level of catabolism (P-*miox*^f01770^/P-*miox*^f01770^). Not surprisingly, the intermediate levels of *myo*-inositol catabolism in the two RNAi knockdown strains *MIOX*i*2*/*+*; *+*/*Act5CGal4-3* and *MIOXi3*/*Act5CGal4-3* showed intermediate levels of *myo*-inositol in larval tissues (Figure 4A). When rich food was supplemented with 50 µM of *myo-*inositol, the *myo-*inositol levels in all the strains increased (Figure 4A). Interestingly, whole *MIOX*i*2*/*+*; *+*/*Act5CGal4-3* and *MIOXi3*/*Act5CGal4-3* larvae fed rich food with 50 µM *myo-*inositol supplementation had more total *myo-*inositol than P-*miox*^f01770^/P-*miox*^f01770^ larvae fed rich food without *myo-*inositol supplementation, yet only P-*miox*^f01770^/P-*miox*^f01770^ displayed morphological defects (Figure 3B). Larvae with hemolymph removed (carcasses) of *MIOXi2*/*+*; *+*/*Act5CGal4-3* and *MIOXi3*/*Act5CGal4-3* fed rich food with 50 µM *myo-*inositol supplementation had lower total *myo-*inositol than carcasses of P-*miox*^f01770^/P-*miox*^f01770^ larvae fed rich food without *myo-*inositol supplementation (Figure 4A). Apparently, adding dietary *myo-*inositol, and by doing so increasing the circulating *myo-*inositol levels, does not affect development but does further reduce obesity (buoyancy and TAG) and hemolymph glucose levels in all the strains (Figure 4B,C). It is tantalizing to speculate that at least some of the developmental defects observed in P-*miox*^f01770^/P-*miox*^f01770^ stem from abnormally high *myo-*inositol levels during embryogenesis prior to the organism feeding. Notably, abnormally high *myo-*inositol levels contribute to the pathology of some human disorders of neurological development and dysfunction [6,7,75,76,77].

Alterations of *myo-*inositol metabolism been implicated in many human diseases and disorders including diabetes, obesity, and hyperglycemia. Low MIOX expression/activity, which should elevate *myo-*inositol levels, rescued mice and rats from renal injury and oxidative stress [27,62]. In this study, low MIOX levels were shown to reduce obesity and hyperglycemia in populations of *D. melanogaster* larvae. In addition, the supplementation of the rich food with 50 µM of *myo-*inositol further reduced obesity and hyperglycemia. In humans, dietary *myo-*inositol augmentation may mitigate obesity and hyperglycemia (high blood glucose) [4,7,78]. These results complement studies which established that reduction in the inositol 1,4,5-trisphosphate receptor (InsP3R), either by knockdown or mutation, resulted in obese adult fruit flies [79]. Moreover, these results are in agreement with studies demonstrating that an increase in *myo-*inositol, via the overexpression of the *myo-*inositol synthetic gene or the addition of dietary *myo-*inositol, decreased obesity and hyperglycemia in *D. melanogaster* [17]. In summary, increased *myo-*inositol, regardless of its source, can mitigate diabetes-associated obesity and hyperglycemia. This study, at the junction of metabolism and development, furthers the understanding of the importance of *myo-*inositol catabolism and the regulation of intracellular *myo-*inositol levels and may have implications for the treatment of diabetes and developmental disorders.

## 4. Materials and Methods

### 4.1. Fly Stocks and Maintenance

Stocks obtained from the Bloomington Drosophila Stock Center (Bloomington, IN, USA) include the Canton-S (#1, CS) strain, w [1118]; PBac{w[+mC] = WH}CG6910[f01770]/ TM6B, Tb[1] (#18471, hereafter identified as P-*miox*^f01770^/Tb), y[1] w[*]; +;P(w[+mC] = Act5C-GAL4)17bFO1/TM6B, Tb[1] (#3954, hereafter identified as Act-Gal4-3/Tb), w[1118]; CyO, P{w[+mC] = FRT(w[+])Tub-PBac\T}2/wg[Sp-1] (#8283), w[*]; TM3, Sb [1] Ser [1]/TM6B, Tb[1] (#2537, hereafter identified as w[*]; Tb/Sb), and w[1118]; Df(3L) Ly, sens(Ly-1)/TM6B, P{w[+mW.hs]=Ubi-GFP.S65T}PAD2, Tb[1] (#4887) and w[1]; sna[Sco]/CyO, P{w[+mC] = GAL4-Hsp70.PB}TR1, P{w[+mC] = UAS-GFP.Y}TR1 (#5702) used to introduce GFP-marked chromosomes 3 and 2, respectively). The two RNAi strains P{KK102548}VIE-260B (#103766, hereafter identified as *MIOX*i2/*MIOX*i2) and w[1118]; P{GD12073} v22464/TM3, Tb (#22464, hereafter identified as *MIOX*i3/Tb) were obtained from the Vienna Drosophila Research Center (Vienna, Austria). The RNAi strain *MIOX*i2/*MIOX*i2 is homozygous for an RNAi construct complementary to the third exon of *CG6910* that was inserted via P-element to chromosome 2. The other RNAi strain, *MIOX*i3/Tb, also containing sequences complementary to the third exon of *CG6910,* is heterozygous for a different RNAi construct that was inserted via a phiC31 sequence to chromosome 3. Both RNAi constructs contain UAS_GAL4_ sequences controlled by GAL4 expression. *MIOX*i2/+; +/ActGal4-3 and *MIOX*i3/ActGal4-3 were generated by mating strains marked with GFP on corresponding balancer chromosomes. The third strain was generated by introducing a GFP-marked balancer third chromosome (TM6) into the P-*miox*^f01770^/Tb, an existing strain with an approximately 7.2kb *piggyBac* WH-element inserted into the second intron of *CG6910*, and non-GFP non-tubby homozygotes (P-*miox*^f01770^/ P-*miox*^f01770^) were then readily identified. The *piggyBac*-element insertion in *CG6910* was remapped and its location confirmed using flanking sequence data [80]. To create a double-marked (Tb/Sb) transposase strain, the strain harboring the transposase (#8283) was crossed to a third chromosome double balancer strain (#2537) introducing the Tb marked TM6B chromosome. These Tb marked flies were again crossed to the third chromosome double balancer strain (#2537) to introduce the Sb marked TM3 chromosome creating the double marked transposase strain w [1118]; CyO, P{w[+mC] = FRT(w[+])Tub-PBac\T}2; TM3, Sb [1] Ser [1]/TM6B, Tb [1] (hereafter identified as w[+]; *piggyBac* transposase; Tb/Sb). To excise the *piggyBac* WH-element inserted in the *MIOX* gene (*CG6910*), the w[+]; *piggyBac* transposase; Tb/Sb strain was crossed to P-*miox*^f01770^/Tb (#18471). According to Thibault et al. 2004 [81], excisions of this *piggyBac* WH-element are precise. Seventy-two single Tubby (Tb) progeny with dark red eyes (double w[+mC]) with curly wings (CyO) harboring both the transposase and the *piggyBac* WH-element were individually mated with w[*]; Tb/Sb. Eight of the seventy-two crosses produced white-eyed, straight-winged progeny, carrying neither the *piggyBac* WH-element (chromosome 3, *CG6910*) nor the transposase (chromosome 2, CyO, P{w[+mC] = FRT(w[+])Tub-PBac\T}2). Of these eight, three produced multiple white-eyed, straight-winged progeny which were used to establish stocks. Three or four individuals from each of the three independent lines (eleven flies) with putative *piggyBac* WH excisions (from the *MIOX* gene (*CG6910*)) were individually crossed to w[*]; Tb/Sb, and Tb progeny were selected.

Flies were maintained in standard laboratory conditions at 25 °C and 70–80% humidity on a 12 h:12 h light–dark cycle. All fly stocks were grown on either rich food (BDSC cornmeal food, https://bdsc.indiana.edu/information/recipes/bloomfood.html (accessed on 29 April 2011)) or modified food (per liter, 10 g of agar (Fisher Scientific, Waltham, MA, USA), 80 g of brewer’s yeast (Genesee), 20 g of yeast extract (Fisher Scientific), 20 g of peptone (Fisher Scientific), and sucrose (Fisher Scientific) as indicated, [49]) with or without 50 µM of *myo-*inositol (Sigma, St. Louis, MO, USA) as indicated, which is sufficient to support growth of a homozygous *Inos* deletion mutant (inos^ΔDF^/inos^ΔDF^) [16]. Semi-defined food (casamino acids sucrose (CAA-S) and casamino acids *myo-*inositol (CAA-I)) was prepared essentially as described [82] with modifications [83]. Briefly, defined food contained 0.4 g of lecithin, 0.613 g of vitamin mix, 5.5 g of casamino acids, 3.15 g of agar, and 7.5 g of sugar (sucrose or *myo-*inositol) or no sugar per 100 mL. The vitamin mix was composed of 3 g of cholesterol, 0.02 g of thiamine, 0.01 g of riboflavin, 0.12 g of nicotinic acid, 0.16 g of calcium pantothenate, 0.25 g of pyridoxine, 0.016 g of biotin, 0.03 g of folic acid, 14 g of NaHCO_3_, 18.3 g of KH_2_PO_4_, 18.9 g of K_2_HPO_4_, and 6.2 g of MgSO_4_. Then, 350 μL of 30% Tegosept was added to the 100 mL of food.

### 4.2. RNA Extraction and qRT-PCR

Total RNA was extracted from 10–20 third-instar larvae or adult flies grown on the food indicated using Trizol^TM^ (Life Technologies, Carlsbad, CA, USA) [84]. Total RNA (1 µg) was DNase-treated using the DNA-free Kit (Ambion, Foster City, CA, USA) with inactivation buffer (DNA-free DNA Removal Kit, Invitrogen, Carlsbad, CA, USA). cDNAs were generated using oligo (dT) 18 primers (Eurofins, Luxembourg), dNTPs (ThermoFisher, Waltham, MA, USA), and Moloney Murine Leukemia Virus Reverse Transcriptase (M-MLV RT) (Fisher, Waltham, MA, USA). After amplification, the samples were treated with RNAse H (New England BioLabs, Ipswich, MA, USA). The cDNA was diluted in RNase/DNase-free water (ThermoFisher) (1:16), and qRT-PCR experiments were performed using ThermoScientific Absolute qPCR Mix, SYBR Green, ROX (Fisher) in an Applied Biosystems StepOnePlus System. Triplicate samples were used in all the experiments including linearizations and melt curves. All the experiments were performed at least three independent times (separate biological samples), as indicated in the figure legends. The results were normalized to the transcript levels of *Drosophila melanogaster* ribosomal protein L32 (RpL32). The following primers were used: *MIOX* exon 2-3 forward GACACCACCGATCCTCTAAAGG and reverse GGAAGGCGTGGATGATGT, *RpL32* forward CCAGCATACAGGCCCAAGAT and reverse GCACTCTGTTGTCGATACCC.

### 4.3. Protein Extraction and the MIOX Activity Assay

The MIOX activity assays were established for *D. melanogaster* based on protocols for hog kidneys and fungi [31,32]. For each sample, ten flies or ten larvae were homogenized in 300 μL of 20 mM sodium acetate (pH 6.0), 2 mM L-cysteine (pH 4.5), 1 mM glutathione (pH 3.5), 1mM ferrous ammonium sulfate (pH 4.5), and 10 μL of protease inhibitor (Halt™ Protease Inhibitor Cocktail (100X), Thermo Scientific). Protein concentrations of the crude lysate cleared supernatants were determined using the Bradford Assay [85] with bovine serum albumin (Pierce™ Bovine Serum Albumin Standard, Thermo Scientific) and using a dye concentrate (5000006, Bio-Rad, Hercules, CA, USA) for the colorimetric analysis. *myo-*Inositol catabolism was assayed in 450 μL of 50 mM sodium acetate (pH 6.0), 2 mM L-cysteine (pH 3.5), 1 mM ferrous ammonium sulfate (pH 4.5), and 60 mM *myo-*inositol (or water) with 15 μg of crude lysate protein. The reactions were incubated at 30 °C. Then, 200 μL aliquots were removed at 0 and 30 min, immediately added to 55 μL of 30% trichloroacetic acid (TCA), and incubated at 100 °C for two minutes. The glucuronic acid concentrations of the cleared supernatants were determined by adding 500 μL of orcinol reagent (0.08 g of orcinol, 0.018 g of FeCl_3_ and 20 mL of concentrated HCl) to 250 μL of the sample and were measured at 660 nm [28].

### 4.4. Survival Studies

Twenty CS or Act-Gal4-3/TbGFP virgin females and ten CS or *MIOX*i2/*MIOX*i2 or *MIOX*i3/TbGFP males were mated on rich food. For each of the six independent trials, twenty pupae from each cross were transferred onto rich food with 0 or 50 µM of *myo-*inositol added, CAA-S, CAA-I, or CAA-0 (no sugar added). Survival was monitored daily.

### 4.5. Pupariation and Eclosion

Female and male adults (2:1) were placed in vials of standard rich food in a 25 °C incubator at 70–80% humidity on a 12 h:12 h light–dark cycle. The progeny (embryos) were sorted using the GFP marker and reconfirmed as larvae. To allow sufficient time for genotypes with developmental delays to eclose, the vials were checked daily for up to 28 days. The number of pupae were recorded, as was the number of adults that eclosed.

### 4.6. Light Microscopy

Fifteen control CS and sixteen independent experimental P-*miox*^f01770^/P-*miox*^f01770^ specimens were viewed on a Nikon SMZ1500 microscope, and images were captured using a Micropublisher 6 color CCD camera system (Teledyne Q imaging, Surrey, BC, Canada).

### 4.7. myo-Inositol Assay

Five third-instar larval carcasses per sample were collected by puncturing five larvae and draining the hemolymph via centrifugation. The samples were homogenized in dH_2_O, and the Megazyme *myo*- inositol assay kit (K-INOSL) was used as per the manufacturer’s instructions. Three independent trials were performed.

### 4.8. Buoyancy Assay

Experiments were conducted essentially as described by Reis [48,54] using 20–30 3rd-instar larvae per sample, with initial results confirmed by the method of Hazegh and Reis [54]. A relationship between the percentage of larvae floating in a buoyancy assay and the percent body fat of the larvae has been established [48,86]. In this study, the relative obesity of a population of larvae is defined as the proportion of the larvae floating in the buoyancy assay.

### 4.9. Triacylglyceride (TAG) Assay

Experiments were performed essentially as previously described [39] and normalized to total protein (using the Bradford Assay described above). Six third-instar larvae per sample were homogenized in 1xPBS with 9.1% Tween, and the Serum Triglyceride Determination Kit (TR0100, Sigma, St. Louis, MO, USA) and Triglyceride Reagent (T2449, Sigma) were used as per the manufacturer’s instructions for three independent trials.

### 4.10. Hemolymph Glucose Assay

Experiments were performed essentially as described by Tennessen et al. [39]. Hemolymph was collected by puncturing five third-instar larvae per replicate, and the Sigma Glucose (GO) Assay Kit GAGO-20 was used. Three independent trials were performed.

### 4.11. Computational Analyses

The National Center for Biotechnology Information’s (NCBI) Basic Local Alignment Search Tool (BLASTP) with default settings (BLOSSUM 62) was used to identify the MIOX homolog in *D. melanogaster*.

### 4.12. Statistical Analyses

Standard error was calculated for all experiments. Mantel–Cox (log rank) tests were performed to calculate significance in survival studies. The *p*-values of pairwise comparisons were determined using Student’s two-tailed *t*-test.

## 5. Conclusions

In the model organism *Drosophila melanogaster,* the elimination of *myo-*inositol catabolism and the associated high levels of *myo-*inositol cause severe developmental defects. A reduction in *myo-*inositol catabolism or dietary *myo-*inositol supplementation yields the beneficial effects of higher *myo-*inositol levels (reduced obesity and hemolymph (blood) glucose in *Drosophila melanogaster* third-instar larvae) without causing developmental defects. This suggests that dietary inositol supplementation may serve as a therapeutic agent.

## Data Availability

Strains available upon request. All the relevant data are within the paper.
